# Explainable artificial intelligence for omics data: a systematic mapping study

**DOI:** 10.1093/bib/bbad453

**Published:** 2023-12-18

**Authors:** Philipp A Toussaint, Florian Leiser, Scott Thiebes, Matthias Schlesner, Benedikt Brors, Ali Sunyaev

**Affiliations:** Department of Economics and Management, Karlsruhe Institute of Technology, Karlsruhe, Germany; HIDSS4Health – Helmholtz Information and Data Science School for Health, Karlsruhe, Heidelberg, Germany; Department of Economics and Management, Karlsruhe Institute of Technology, Karlsruhe, Germany; Department of Economics and Management, Karlsruhe Institute of Technology, Karlsruhe, Germany; Biomedical Informatics, Data Mining and Data Analytics, Faculty of Applied Computer Science and Medical Faculty, University of Augsburg, Augsburg, Germany; Division of Applied Bioinformatics, German Cancer Research Center (DKFZ), Heidelberg, Germany; Translational Oncology, National Center for Tumor Diseases, German Cancer Research Center (DKFZ), Heidelberg, Germany; Department of Economics and Management, Karlsruhe Institute of Technology, Karlsruhe, Germany

**Keywords:** explainable artificial intelligence, omics, biomedical data, machine learning, interpretable artificial intelligence, systematic mapping study

## Abstract

Researchers increasingly turn to explainable artificial intelligence (XAI) to analyze omics data and gain insights into the underlying biological processes. Yet, given the interdisciplinary nature of the field, many findings have only been shared in their respective research community. An overview of XAI for omics data is needed to highlight promising approaches and help detect common issues. Toward this end, we conducted a systematic mapping study. To identify relevant literature, we queried Scopus, PubMed, Web of Science, BioRxiv, MedRxiv and arXiv. Based on keywording, we developed a coding scheme with 10 facets regarding the studies’ AI methods, explainability methods and omics data. Our mapping study resulted in 405 included papers published between 2010 and 2023. The inspected papers analyze DNA-based (mostly genomic), transcriptomic, proteomic or metabolomic data by means of neural networks, tree-based methods, statistical methods and further AI methods. The preferred post-hoc explainability methods are feature relevance (*n* = 166) and visual explanation (*n* = 52), while papers using interpretable approaches often resort to the use of transparent models (*n* = 83) or architecture modifications (*n* = 72). With many research gaps still apparent for XAI for omics data, we deduced eight research directions and discuss their potential for the field. We also provide exemplary research questions for each direction. Many problems with the adoption of XAI for omics data in clinical practice are yet to be resolved. This systematic mapping study outlines extant research on the topic and provides research directions for researchers and practitioners.

## INTRODUCTION

With the ever-increasing availability of biomedical data, novel and more advanced data analysis approaches, especially artificial intelligence (AI) and its sub-areas of machine learning (ML) and deep learning (DL), have emerged [1–3]. Biomedical data range from patient information (e.g. date of birth, sex) and routine medical data (e.g. height, weight, blood pressure) to more specialized laboratory data (e.g. genetic data, proteins, metabolites) [4, 5]. In particular, laboratory data from the omics sciences have gained widespread interest among researchers in recent years due to their complexity, availability and potential for generating new medical insights (i.e. understanding underlying biological processes) [6, 7].

While AI models offer improved predictive performance compared to traditional expert analysis, the high dimensionality of omics data results in highly complex models with their inner workings and outputs being opaque to humans (black box models) [8, 9]. Consequently, AI models for omics data analysis are often hard to interpret and lack understandability [4, 10]. Understanding the inner workings of black box models, however, is necessary to justify AI-based decisions [11, 12]. Especially in healthcare, where these decisions affect human lives, experts require far more information than simple binary decisions (e.g. cancer being present or not) to support a diagnosis or treatment recommendation [13, 14]. Additionally, our lack of understanding regarding the biological processes from which omics data emerge makes understanding an AI model, its inner workings and its outputs evermore important [6].

Building on the need for understandability and transparency of AI models, research on explainable AI (XAI) has surged recently [4, 15, 16]. XAI research aims at producing more human-understandable AI models while maintaining or improving their performance (e.g. accuracy, precision, runtime) [4, 17, 18]. To achieve this, XAI offers two main paradigms. For one, researchers aim to augment black box models into interpretable (i.e. transparent) models, for example, through simplification or architecture modification [10, 19]. For another, AI models can be extended with post-hoc explanations, which are implemented to clarify or detail their internal functions, such as feature relevance or visual explanations [10, 11].

Although XAI research has shown the potential to increase understandability, for example, through providing insights into the underlying biological processes, many problems of applying AI in clinical practice are still unresolved due to open medical, legal, ethical and societal questions, even though they promise better results [11, 14, 20, 21].

## OBJECTIVES

Recently, various research streams investigating the topic of XAI for omics data have emerged. Coming from diverse viewpoints, research areas applying XAI to omics data include but are not limited to the information and computing sciences (e.g. [22]), the biomedical and clinical sciences (e.g. [23]), the natural sciences (e.g. [24, 25]) and engineering (e.g. [26]). Because many of the omics communities operate isolated on their specific data, innovative XAI advances and helpful findings are often not transferred. This has resulted in a scattered literature landscape on the application of XAI for omics data.

When broadening our view to the biomedical or healthcare domain in general, there are some reviews (e.g. [16, 21, 27]) that capture current use cases of XAI for biomedical and healthcare data, as well as the ethical and legal debate surrounding the topic. However, research that synthesizes the scattered literature on XAI for omics data is scarce. While there exist some general reviews on AI/ML for omics data (e.g. [28, 29]), most reviews in this area focus on a single omics field, like genomics (e.g. [20, 30]), proteomics (e.g. [31]), microbiomics (e.g. [32]) or metabolomics (e.g. [33]) and single omics data types, such as single-nucleotide polymorphism (SNP) data (e.g. [34]), 3D-genomic data (e.g. [35]) or ribonucleic acid (RNA) sequence data (e.g. [36, 37]). Other reviews focus on AI research within typical omics data use cases such as oncology (e.g. [38, 39]) and gene expression analysis (e.g. [40]). More importantly, however, most of these studies do not focus on explainability of omics data analysis but, if at all, merely call for more research on the topic. Reviews with a focus on XAI for omics data are few and far between and limited to specific subareas. For example, Novakovsky *et al*. [41] as well as Talukder *et al*. [42] review the current use of explainability methods on DL while only considering genomic data. Further, Miotto *et al*. [7] present a review with a narrow focus on DL methods for biomedical data and healthcare in general, highlighting opportunities and challenges, including the use of XAI.

Given the interdisciplinary nature of the topic, the scattered landscape of extant literature, the lack of omics sciences-traversing reviews and thus, shortcomings of existing knowledge on XAI for omics data, an overview of the existing literature on XAI for omics data is sorely needed. Such an overview can provide insights into the isolated advances in the omics sciences, highlight promising XAI approaches and help detect common issues in applying XAI to omics data. Additionally, with multi-omics data analysis rapidly gaining in importance for healthcare [43], an overarching understanding of the current state of XAI for omics data in a systematic manner is required to enable targeted future research across multiple omics sciences. Consequently, our study aims to answer the following research questions (RQs):

RQ1: How does extant literature apply XAI to omics data?
RQ2: What are possible future research directions of XAI for omics data?

## METHODS

**Figure 1:**
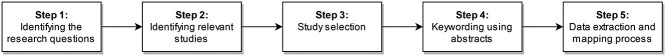
Overview of the systematic mapping process adapted from Petersen [48].

For our review, we chose the systematic mapping approach because it is established in medical research and provides a well-defined methodology (e.g. [44–47]). It entails the development of a classification scheme and subsequent classification of relevant literature. Toward that end, we followed the five-step procedure by Petersen [48] and adapted it to our needs (Figure 1). Hereafter, we briefly explain each step.

### Identifying relevant studies

Having identified the relevant research questions, we first conducted a search of available literature. To find all available literature concerning XAI for omics data, we specified a search string representing the three important aspects of this research: explainability, AI and omics data. With respect to the interdisciplinary nature of this research field, we investigated multiple databases, namely, Scopus, Web of Science and PubMed, as well as the preprint databases arXiv, MedRxiv and BioRxiv. Our initial search, limited to title, abstract and keywords, was conducted on 7 May 2023 and resulted in a set of 6234 publications. Table 1 summarizes our search strategy, including the detailed search string.

**Table 1:** Overview of the search strategy.

Search string	(explainab^*^ OR interpretab^*^) AND (‘artificial intelligence’ OR ‘machine learning’ OR ‘deep learning’) AND (^*^omic^*^ OR biomedical OR ‘life science’ OR genom^*^ OR genetic^*^ OR metagenom^*^ OR neurogenom^*^ OR pangenom^*^ OR epigenom^*^ OR lipidom^*^ OR proteom^*^ OR glycom^*^ OR foodom^*^ OR transcriptom^*^ OR metabolom^*^ OR nutrigenetic^*^ OR nutrigenomic^*^ OR pharmacogenomic^*^ OR pharmacomicrobiomic^*^ OR toxicogenomic^*^)
Fields	Title; abstract; keywords
Databases	Regular databases: Scopus, PubMed, Web of Science Preprint databases: BioRxiv, MedRxiv, arXiv
Publication types	Journal articles, conference papers, preprints
Date range	Peer-reviewed publications: January 2010 to May 2023 Preprints: January 2017 to May 2023

### Study selection

From this initial set, each publication was assessed for relevance to our topic. After removing 2355 duplicates based on title, DOI and authors, we excluded publications before 2010 (*n* = 133) and preprints if they were published before 2017 (*n* = 6). This left us with 3740 potentially relevant publications.

Next, three researchers assessed the eligibility of the publications based on title, abstract and keywords with predetermined inclusion and exclusion criteria. For the regular databases, we included only peer-reviewed full-paper articles published in scientific journals or conferences. Studies should focus on the application of AI or XAI methods for omics data. We thus excluded publications with no or weak focus on omics data (*n* = 1826), those with no focus on XAI (*n* = 571) and those with no focus on either (i.e. off-topic; *n* = 348). If the assessments differed, the respective publications were discussed among the three researchers to reach an agreement. Further, papers that were not articles, such as conference summaries or not peer-reviewed manuscripts, were excluded (*n* = 203). A non-English publication (*n* = 1) was also excluded.

**Figure 2:**
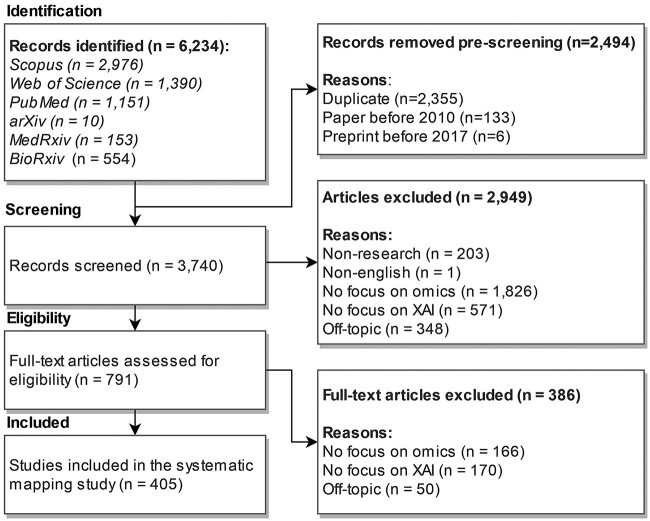
PRISMA flow diagram of the study selection process.

Last, the same researchers analyzed the full text of the remaining 791 publications for eligibility. During this full-text analysis, we again excluded publications with no focus on XAI (*n* = 170), omics data (*n* = 166) or both (*n* = 50) that were not apparent by assessing title, abstract and keywords. Therefore, we included 405 publications in the systematic mapping process. The study selection process is depicted in Figure 2. A full list of relevant studies is included in Supplementary Material 1.

### Keywording using abstracts

The next step was to develop a classification scheme by utilizing keywording. For this, a subset of the included studies’ abstracts was sampled and searched for keywords. These keywords were then used to deduct relevant facets for the topic of interest. Due to the interdisciplinary nature of this study, the classification scheme needed to represent dimensions regarding AI, XAI and omics data. Three researchers each analyzed the abstracts of 45 papers (11.1%) for keywords, from which we then formed 10 facets in a discussion among all authors. Additionally, we also consulted scientific literature to determine possible characteristics (i.e. the manifestations of a facet). Table 2 provides an overview of the classification scheme, including an explanation for each facet. A more detailed explanation of the classification scheme development, together with all possible categories (manifestations) per facet and a detailed overview of different explainability methods, can be found in Supplementary Material 2.

**Table 2:** Developed classification scheme with 10 facets.

**Facet**	**Explanation**
AI method	Learning architecture/algorithm used
AI task	Type of task the AI model performs
Model approach	Whether a new model is proposed or an existing model used
XAI model	Whether the model is directly interpretable or post-hoc explainable
Explainability method	Which explainability technique the XAI model uses
XAI generalizability	Whether the XAI approach can be transferred to other AI methods
Omics field	Concerned discipline from the omics sciences
Omics data	Input data for the XAI model
Medical use case	The specific use case that the XAI model aims to solve
Medical field	The medical field in which the XAI model is applied

### Data extraction and mapping process

The last step was the classification of all included studies according to the classification scheme. Following an initial independent coding and subsequent discussion of 20 papers by three researchers to ensure the same understanding of the classification scheme and task at hand, the remaining papers were single-coded by one researcher each. The complete coding of all 405 papers was then discussed with all authors and further used to collate and chart systematic maps as well as further analyses. We present the main findings resulting from this process in the following section. Additional charts can be found in Supplementary Material 3. A glossary of abbreviations used in this article can be found in Supplementary Material 4.

## RESULTS

### Descriptive results

**Figure 3:**
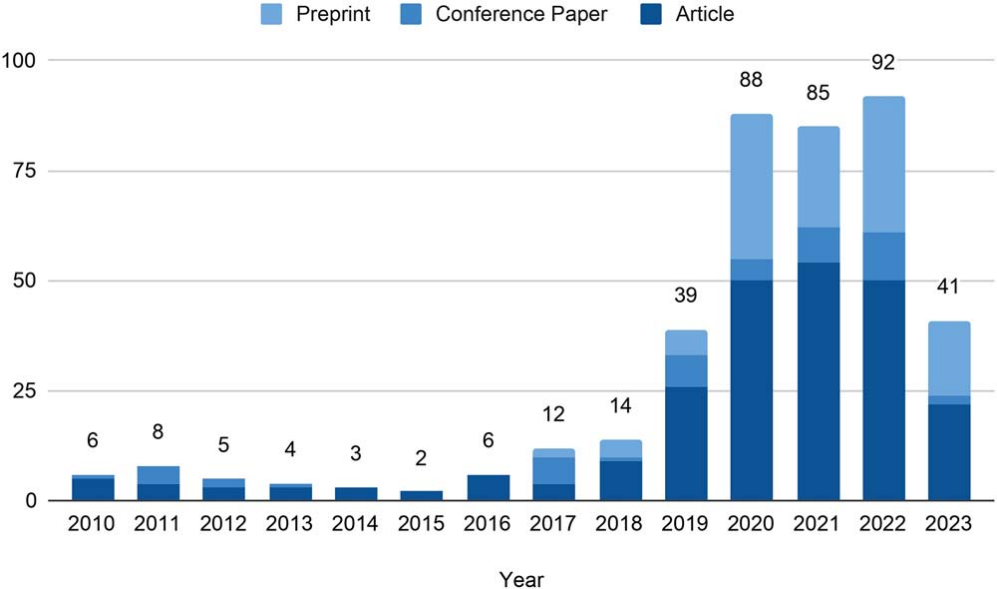
Number and document type of publications by year.

**Figure 4:**
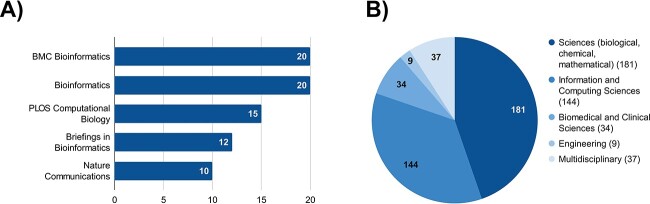
Top five outlets (**A**) and scientific fields (**B**) of included publications.

We included 405 studies in this systematic mapping study, all published after 2010. Being below 10 prior to 2017, the number of publications per year has continuously grown from 12 to 92 studies in 2022, with 2023 likely to achieve similar numbers (Figure 3). The studies are published in 137 outlets, with the top five outlets accounting for over 40% of all publications. The majority of the manuscripts are published in preprint databases. BioRxiv alone accounts for 102 (25.2%) of all studies, and MedRxiv holds 13 (3.2%) relevant publications. Excluding the preprint databases, biomedical outlets like BMC Bioinformatics (*n* = 20), Bioinformatics (*n* = 20), PLOS Computational Biology (*n* = 15), Briefings in Bioinformatics (*n* = 12) and Nature Communications (*n* = 10) are most prevalent (Figure 4A). 95 out of the 137 outlets have only published one relevant study. Examining the scientific fields, the majority of studies (*n* = 181) emerge from the sciences (i.e. mostly biology-, chemistry- or mathematics-related outlets), while 144 stem from the information and computing science disciplines. Also, 34 studies can be attributed to the biomedical and clinical sciences, as well as nine studies to the field of engineering (Figure 4B). Lastly, 37 publications have a multidisciplinary background encompassing at least two of the prior scientific fields. Although all included studies follow a design approach (i.e. design, implementation and evaluation of specific AI models), four studies [49–52] also conduct an extensive literature review and/or case study, qualifying them as mixed methods approaches. 247 studies only provide an implementation or prototype, while 158 develop a general concept in addition to implementation. Lastly, slightly more than half of the studies (*n* = 225) invent new model approaches, while the remaining 180 studies rely on existing model approaches.

**Figure 5:**
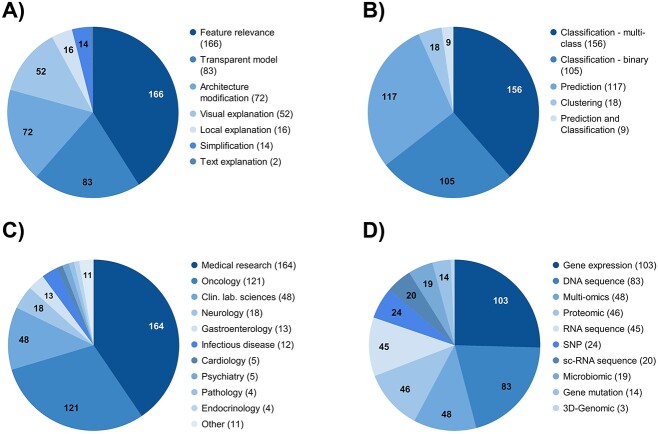
Distribution of explainability method (**A**), AI task (**B**), medical field (**C**) and omics data (**D**) of included publications.

As seen in Figure 5A, the most used explainability method is feature relevance (*n* = 166), followed by the use of inherently transparent models (*n* = 83) and applying architecture modification (*n* = 72). Visual explanations are utilized in 52 studies, while local explanations and simplifications are only employed 16 and 14 times, respectively. We also identified two studies providing text explanations. Regarding the AI tasks (Figure 5B), multi-class classification (*n* = 156) and binary classification (*n* = 105) predominate. Prediction tasks are performed in 117 studies, while nine other studies also conduct classification. Lastly, 18 papers carry out clustering.

The studies can be attributed to 17 different medical fields (Figure 5C). The three largest are medical research (*n* = 164), oncology (*n* = 121) and clinical laboratory sciences (*n* = 48). All subsequent categories were identified for 18 studies or fewer. As for the omics data (Figure 5D), gene expression data are most frequently used in the considered studies (*n* = 103), followed by DNA sequence data (*n* = 83) and multi-omics data (*n* = 48), which use a combination of at least two data categories. 46 studies use proteomic data, and 45 studies analyze RNA sequence data. Further, the least used data types are SNP data (*n* = 24), single-cell ribonucleic acid (sc-RNA) sequence data (*n* = 20), microbiomic data (*n* = 19), gene mutation data (*n* = 14) and 3D-genomics data (*n* = 3).

**Figure 6:**
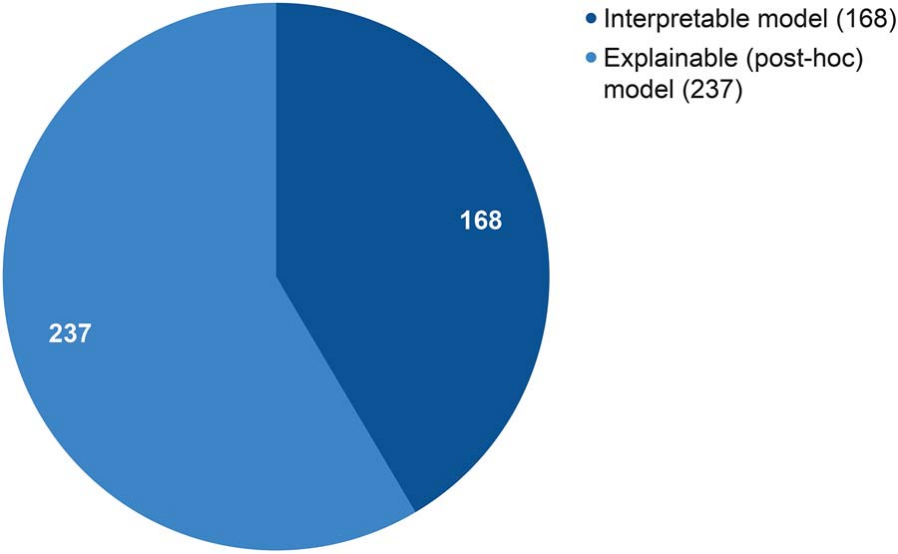
Distribution of XAI models found in our literature set.

Further, the included studies can be divided into two categories for the XAI model used (Figure 6). In total, 168 studies designed an interpretable model, while 237 opted for an explainable model (i.e. post-hoc procedures). It should be noted that 72 of the studies coded as interpretable models achieve interpretability through architecture modification or simplification. As for post-hoc explainable XAI models, 44 papers have underlying transparent AI models but nonetheless apply post-hoc explainability procedures to improve understandability. Additionally, 19 studies developing new interpretable models also added post-hoc explainability methods to their otherwise transparent models.

### AI methods used for omics data

**Figure 7:**
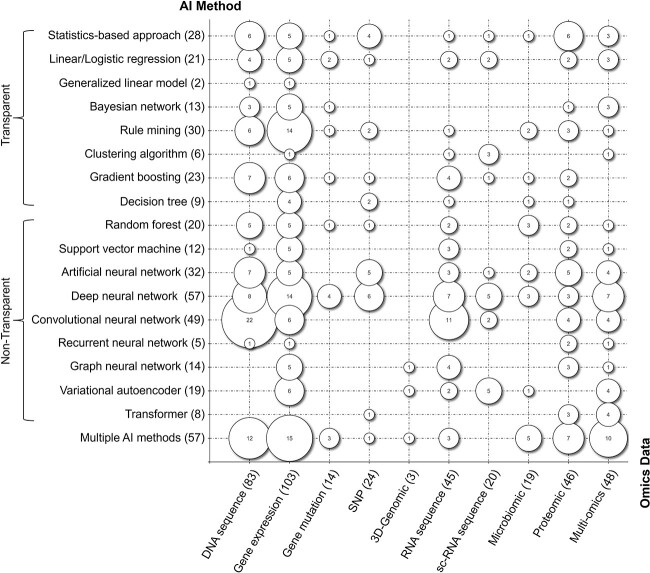
Bubble chart of applied AI method and used omics data.

Combinations of multiple facets can be visualized and analyzed as bubble charts. We first combine the applied AI method with the underlying omics data to investigate which data and AI method combinations have been considered for use with XAI (Figure 7). These charts outlay the number of studies that each share a specific combination of categories (e.g. six papers used a statistics-based approach as the AI method and DNA sequence as their omics data). Supplementary Material 2 provides a detailed explanation of the different AI methods.

#### DNA-based omics data

DNA sequence data are the second most used omics data in our review (*n* = 83). Therefore, it is not surprising that many different AI methods are applied for various analyses. Some approaches use models generally considered transparent, like linear/logistic regression (*n* = 4), generalized linear models (GLMs; *n* = 1), gradient boosting (*n* = 7) or rule mining (*n* = 6). However, the majority of studies (53.0%) focus on non-transparent models, including random forests (*n* = 5), support vector machines (SVMs; *n* = 1) and neural networks (NNs; *n* = 38). In fact, DNA sequences analyzed by means of convolution neural networks (CNNs) are the most common combination overall, with 22 instances (5.4%). For example, Zuallaert *et al*. [53] utilize a CNN for splice site prediction on DNA sequence data. The found motifs are then visualized for the explainability of the network.

With 103 studies (25.4%), gene expressions are the most used omics data type in our study. Each AI method, with the exception of transformers, has been implemented at least once for gene expression data. Nonetheless, the most prominent approaches are multiple AI methods (*n* = 15), rule mining (*n* = 14) and deep neural networks (DNNs; *n* = 14). For example, Shams *et al*. [54] develop REM, a rule extraction methodology, by applying rule mining to DNNs and decision trees. Anguita-Ruiz *et al*. [55] apply rule mining to deduce temporal patterns in gene expression data of obesity patients, while Calvo-Dmgz *et al*. [56] build a knowledge and feature selection enhanced rule mining model for cancer type classification. An example of the use of DNNs is the study by Tang and Gottlieb [57], who predict the drug sensitivity of cancer patients from gene expression data. To explain their DNN, they apply the feature relevance method SHapley Additive exPlanations, commonly known as SHAP.

Per our literature set, gene mutation data have been analyzed rather sparsely with XAI (*n* = 14). Cramer *et al*. [58] use linear regression to predict the drug response of cancer patients, whereas Sah *et al*. [59] apply a logistic regression for cancer classification, and Fuji [60] as well as Warrell *et al*. [61] utilize a DNN for pathogenicity and cancer type prediction, respectively.

We also identified 24 studies (5.9%) focusing on SNP data. Most studies here utilize a DNN (*n* = 6) or shallow artificial neural network (ANN; *n* = 5) as their AI method. For example, Sun *et al*. [62] build a DNN to predict eye disease progression from SNP data. Notably, Reyes *et al*. [63] develop a transformer to discover SNP–SNP interactions for Parkinson’s disease. Most remaining papers focus on transparent models (41.7%), such as statistics-based approaches (*n* = 4), decision trees (*n* = 2) or rule mining (*n* = 2).

Our review also resulted in three studies analyzing 3D-genomic data, of which two utilize NNs for Hi-C maps. Highsmith and Cheng [64] design an adversarial network that is capable of enhancing these maps for chromosome structure detection, while Bigness *et al*. [65] use a GNN to deduce gene expressions. Xi and Beer [66], on the other hand, use a multiple AI method pipeline to predict CTCF gene interactions.

#### Transcriptomic data

In total, 45 studies investigate RNA sequence data. While some use transparent models such as gradient boosting (e.g. [67]), clustering algorithm (e.g. [68]) or rule mining (e.g. [22]), the majority of papers (71.1%) present a non-transparent approach such as CNNs (*n* = 11), DNNs (*n* = 7) or GNNs (*n* = 4). We also found three studies like Pan and Shen [69], which utilize multiple AI methods, in particular, a CNN and a deep belief network, to classify binding site motifs from RNA sequences with their iDeep prototype.

Further, 20 studies focus on sc-RNA sequences (4.9%). These do not use any other AI methods compared to general RNA sequence analyses. Most studies apply DNNs (*n* = 5) or variational autoencoders (*n* = 5). For example, Seninge *et al*. [70] present a variational autoencoder for the analysis of sc-RNA sequence data.

#### Microbiomic, proteomic and multi-omics data

Another seldomly analyzed data type is microbiomic data (*n* = 19). The most popular approach for our set is using multiple AI methods (*n* = 5), followed by DNNs (*n* = 3) and random forests (*n* = 3). For example, Yang and Zou [71] utilize an AutoML pipeline, where the model chooses the most suitable AI method based on the provided microbiomic input data and specific classification task. Five studies utilize transparent models, including rule mining (*n* = 2), gradient boosting (*n* = 1) and decision trees (*n* = 1).

With 46 studies, proteomic data are the fourth most analyzed data type of our review. While multiple AI methods (*n* = 7) (e.g. [72, 73]) and statistics-based approaches (*n* = 6) are predominant (e.g. [74, 75]), most methods are implemented by at least one paper. We further identified three studies utilizing transformers, such as Sokhansanj *et al*. [76], aiming to predict COVID-19 infection severity.

Lastly, we also identified 48 studies (11.9%) using multi-omics data. These papers usually argue that one omics data type is not sufficient to achieve their results and thus apply a second data type for additional input information. The applied AI methods are fairly diverse. In fact, multiple AI methods is the most used AI method for multi-omics data (*n* = 10). While some studies, such as Liu *et al*. [77], strive to combine multiple AI methods into one linear model pipeline (e.g. first CNN and then RNN), the majority of these studies actually compare different AI models, which also require different data types (i.e. one AI method is only used for one omics data type). For example, Xu *et al*. [78] compare NNs and tree-based methods with RNA sequence and proteomic data to predict protein abundance. DNNs follow second with seven studies (14.6%). For example, Levy-Jurgenson *et al*. [79] use DNA sequence and gene expression data to predict DNA methylation. Their DNN architecture is modified with an attention mechanism to gain insights into the model. It should also be noted that multi-omics is the only data type where every non-transparent AI method in our coding scheme, including transformers (*n* = 4), has been applied.

### Explainability methods used for AI methods

**Figure 8:**
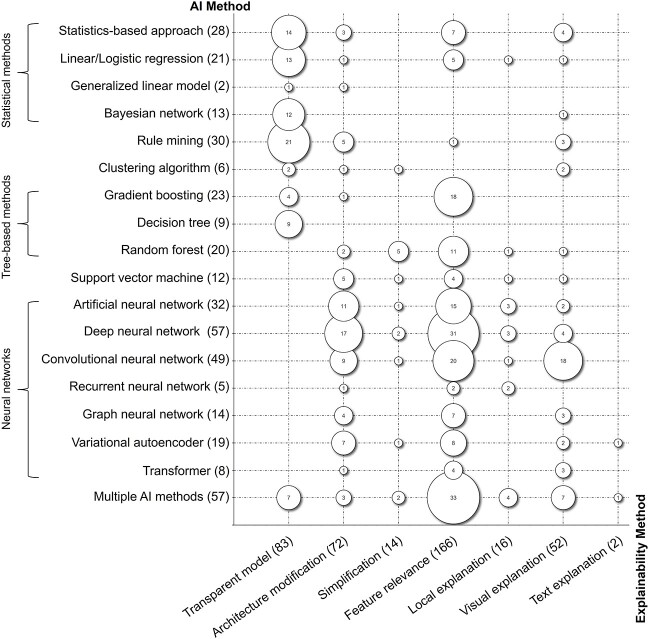
Bubble chart of applied AI method and explainability method.

Having investigated the use of AI methods on different types of omics data, we subsequently analyze the applied explainability methods for the different AI methods. Figure 8 provides a bubble chart of all explainability methods used on certain AI methods to visualize the frequency of the combinations found in our literature set.

#### Neural networks

We found 184 (45.4%) articles using explainability methods on NNs to analyze omics data, the overall most used AI method. Therein, DNNs (*n* = 57) and CNNs (*n* = 49) are predominant. Across all NNs, feature relevance is applied in 87 studies (47.3%). Many approaches utilize SHAP values to identify the feature relevance of the NNs, like Tang and Gottlieb [57], who use a DNN on gene expression data to predict the drug sensitivity of cancer patients. A more sophisticated approach was conducted by Shrikumar *et al*. [80], who develop a framework called DeepLIFT to analyze the feature relevance of DNNs. To validate their framework, the authors utilize DNA sequence data. Next to the 31 manuscripts applying feature relevance on DNNs, 20 and 15 manuscripts use feature relevance for CNNs and ANNs, respectively. For example, Sabando *et al*. [81] apply post-hoc feature analysis to support the prediction of cytochrome interactions.

50 articles (27.2%) apply architecture modification on NNs to achieve interpretability. These architecture modifications change the network layout to represent biological connections like Fuji *et al*. [60], who include knowledge graphs in DNNs to infer genetic mutations. Besides DNNs (*n* = 17), these modifications are especially present in ANNs (*n* = 11) and CNNs (*n* = 9).

An emerging variation of NNs are variational autoencoders (*n* = 19) and transformers (*n* = 8), which mostly use feature relevance or architecture modifications. One especially promising approach is VEGA, a variational autoencoder architecture with enhanced transparency provided by including gene annotations [82]. Transformers also build on visual explanations (*n* = 3, 37.5%), for example, when diagnosing Parkinson’s disease [63].

The most frequent AI method using visual explanations are CNNs (*n* = 18, 36.7%). For example, Ghanbari and Ohler [83] provide consensus motifs to represent the attribution of different binding sites. Visual explanations are used for other NNs as well but less frequently (DNN = 4, GNN = 3, transformer = 3).

#### Tree-based methods

The second group in our mapping applies explainability methods to tree-based methods. According to the taxonomy of Barredo Arrieta *et al*. [10], decision trees are transparent models. Hence, all nine articles implementing decision trees do not apply additional explainability methods.

For other tree-based methods, a common post-hoc approach is feature relevance, for instance, random forests (55.0%) and gradient boosting (78.3%). One article develops an explainability framework to analyze highly multiplexed spatial data in the context of spatial gene expression profiles in a breast cancer data set [84].

For five studies (25.0%), explainability is improved by simplifying random forests. Pliakos and Vens [85] develop an approach to aggregate an ensemble of bi-clustering random forests to fit into one tree, preserving interpretability.

#### Statistical methods

In total, 64 articles apply statistical methods to omics data. 40 articles rely on transparent models (62.5%) like Bayesian networks and linear or logistic regression and do not apply additional post-hoc approaches. While 12 out of 13 articles using Bayesian networks focus on their transparent nature, other transparent methods, like logistic regression, include additional explainability methods such as feature relevance. In all five identified articles using feature relevance on logistic regression algorithms, the feature relevance was conducted post-hoc to increase the explainability of the results after the regression was completed.

Other statistics-based approaches apply architecture modification (*n* = 3), like Alexander and Lange [86], who adapt the loss function of the existing ADMIXTURE algorithm to improve predictions of individual ancestry estimation or feature relevance (*n* = 7), like Pai *et al*. [24] who use patient similarity networks for cancer subtype classification.

#### Further AI methods

Rule mining approaches (*n* = 30) are the third most used AI method besides NNs (*n* = 184) and manuscripts using multiple AI methods (*n* = 57). 21 articles considered rule mining approaches as transparent (70.0%). However, despite their transparent nature, nine articles apply additional explainability measures by visual explanation (10.0%) or architecture modification (16.7%). For example, biological knowledge of DNA gene expressions improved the predictive performance of rule mining approaches by adapting the model architecture to suit this knowledge [56].

Six articles apply different explainability approaches to clustering algorithms. One of the two transparent clustering algorithms includes non-negative decompositions, which results in sparser clustering for cancer detection [87]. Architecture modification helped another approach to build hierarchical clustering based on different data sets [68]. The other three articles increase explainability by applying simplification or visual explanation.

Further, we also found 12 articles applying explainability methods to SVMs. The most prominent method is architecture modification (41.7%). For example, Johannes *et al*. [88] iteratively remove low-impact features for improved interpretability while maintaining high predictive performance for cancer risk stratification. Additionally, four articles apply feature relevance to SVMs, for example, by conducting an additional clustering step to assess feature relevance [89].

In our sample, a large share of articles applies multiple AI methods (*n* = 57). Most of these articles use feature relevance methods (57.9%) like SHAP to assess and compare the explainability of different AI methods (e.g. [90–92]). Other manuscripts compare transparent models (*n* = 7) or support the investigation by visual explanations (*n* = 7), as in Chen *et al*. [93], who develop a framework to compare multiple AI methods.

### Relationships between omics data, ai method and explainability method

**Figure 9:**
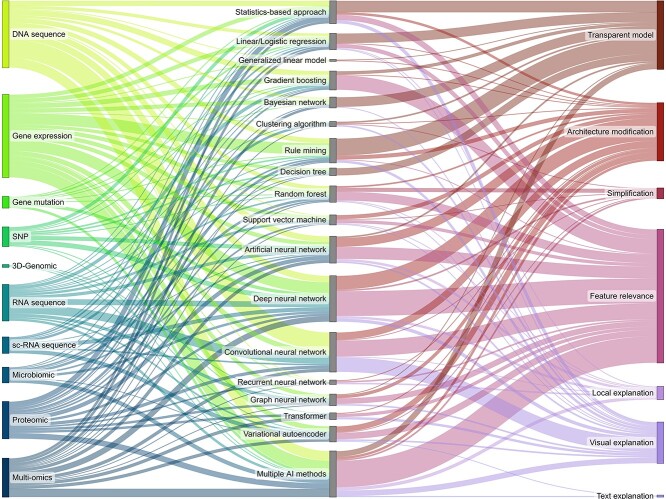
Alluvial chart mapping omics data on AI method and explainability method.

The alluvial chart in Figure 9 combines the insights from our two-dimensional mappings shown in Figures 7 and 8 and unveils an overview of the connections of the investigated mapping dimensions. With the different omics types shown on the left side, we see the heavy focus in the literature on DNA-based data like DNA sequence data and gene expression data. The central column shows the AI methods with large use of DNNs, CNNs and multiple AI methods. The column on the right-hand side shows the applied explainability methods to the corresponding AI methods. Therein, we see the predominant use of feature relevance as well as the limited use of simplification, text explanations or local explanations.

From this overview, we can identify the frequently used AI methods. Hence, we see the use of CNNs for DNA sequence data, rule mining and DNNs for gene expression data and multiple AI methods for multi-omics data. Across all other omics data types, no clear indications of a predominantly used AI approach can be made.

On the right side, we see that most AI method and explainability method combinations are present. There are, however, a few combinations that stand out. For example, most rule mining, Bayesian network and statistics-based approaches are considered transparent models. Nevertheless, a few approaches also apply visual explanations or architecture modifications. DNNs and multiple AI methods are frequently explained by applying feature relevance or architecture modification. While both are also present in papers using CNNs, the focus of explainability methods in CNNs lies with visual explanations. Most gradient boosting algorithms rely on feature relevance to improve the model’s explainability.

## DISCUSSION

Analysis of our systematic maps outlines not only current research trends of XAI for omics data but also reveals important research gaps. In particular, the bubble charts can be used to identify potential research gaps through the absence of certain facet combinations (e.g. ANN, CNN or RNN and gene mutation data). Moreover, sparse combinations may indicate new and upcoming trends (e.g. CNNs and simplification). We also utilized the findings of the alluvial chart (Figure 9) and our thematic insights gained during the classification scheme development and literature classification to deduce possible future research directions. Hence, we present and discuss eight directions for future research that emerged as a result of our literature review. For each direction, we also present illustrative RQs to motivate future investigation and provide actionable guidance for further research. These exemplary RQs are intended to serve as starting points, and future research should expand on them as well as our research directions in general to develop additional RQs relevant to their specific XAI and omics data research projects. Table 3 provides an overview of the research directions as well as derived potential RQs.

**Table 3:** Summary of research directions and illustrative research questions.

**Research direction**	**Illustrative RQs**
A. Apply XAI-supported neural networks to omics data	RQ A-1: How can XAI-supported NNs (especially transformers) be utilized to analyze omics data? RQ A-2: Which data preparations are necessary to apply XAI-enhanced CNNs to omics data? RQ A-3: Which XAI approaches are the most promising for NNs applied to omics data to understand the underlying biological processes?
B. Add post-hoc analysis to transparent models	RQ B-1: How can post-hoc explanations improve the understandability of transparent models for omics data? RQ B-2: How can visual explanations aid the analysis of omics data with transparent AI models?
C. Use simplification and local explanations for post-hoc analysis of omics data	RQ C-1: How can NNs be simplified to improve the transparency of omics data analysis? RQ C-2: How can DL models (such as CNNs) for omics data be explained through local explanations?
D. Develop new interpretable models for omics data and adapt existing post-hoc explainability methods	RQ D-1: Which measures are necessary to construct post-hoc explainable models with omics data that are deployable in healthcare practice? RQ D-2: How can current non-transparent models be modified (e.g. through architecture modification) into novel interpretable models?
E. Combine different explainability methods	RQ E-1: How can different explainability methods be combined for AI-based omics data analysis?
F. Apply explanations by example and text explanations to omics data	RQ F-1: How can explanations by example improve post-hoc analysis of omics data? RQ F-2: How can text explanations improve post-hoc analysis of omics data?
G. Investigate XAI for novel omics data	RQ G-1: How can XAI foster analysis of novel omics data?
H. Explore the role of XAI for omics data in decision-making processes	RQ H-1: How does XAI afford (bio)medical experts to gain new insights, and how does it impact experts’ decision-making in omics use cases?

### Apply XAI-supported neural networks to omics data

Within our dataset, the most frequent AI models are NNs. Every examined data type has at least one variation of an NN approach where explainability methods were included. There are many different types of NNs, ranging from relatively simple three-layer ANNs, over multi-layer DNNs, to specialized NNs such as variational autoencoders, RNNs or transformers. Since all NNs share common architectural features, we believe that research and practice could benefit from applying explainability approaches across NNs. For example, motif extraction, as presented by Amilpur and Bhukya [94], could benefit not only GNNs but also other approaches like RNNs or DNNs with only minor adaptations. Other papers propose the development of simplified interpretable NNs, for example, by including prior knowledge into the models so each node represents biological knowledge [95]. This interpretability approach could also be introduced into DNNs, which might produce even better results due to their increased number of connections. Because NNs belong to the most complex AI methods, they usually lack transparency and understandability. Recently, research has also focused on architecture modification to create knowledge-enhanced and more transparent NNs, like Nguyen *et al*. [96], who include a novel monotonicity constraint between the layers of their DNN.

The frequent use of DL also comes with challenges when analyzing omics data. As illustrated by the above-mentioned examples, there are potential approaches to include explainability methods in NNs. However, the number of studies developing NNs without attempting to understand their inner workings is far greater, and not all explainability approaches are equally well suited for different omics use cases and different NNs. For example, CNNs are mainly applied to DNA and RNA sequence data. A starting point for further research might be to look into other types of sequence data, such as sc-RNA sequence data. Other studies have demonstrated the applicability of DNNs or transformers to gene mutation and SNP data, indicating that other NNs may also be suitable. Especially the latter have been gaining momentum within AI research. However, our review resulted in only eight studies applying transformers to omics data. Nonetheless, four of these studies focus on multi-omics data (e.g. Tang *et al*. [97] include DNA and RNA sequence data as well as proteomic data), highlighting the versatility and applicability of transformers to other omics data. Because NNs are among the most popular and best-performing AI methods for omics data analysis, despite the current lack of understandability, we pose the potential research question:

RQ A-1: How can XAI-supported NNs (especially transformers) be utilized to analyze omics data?

While CNNs can be used for one-dimensional data such as DNA sequence data, their strength lies in the analysis of two-dimensional input data in particular images [10]. In fact, our mapping has shown that many studies adapt their omics data into two-dimensional data. For example, Karim *et al*. [98] utilize the DeepInsight framework to transform their gene expression data into images, which are then classified with a CNN, allowing for visual post-hoc explanations via SHAP pixel maps. The main advantage of CNNs and image input data is that they allow for a visual explanation, which is possibly the best comprehensible explanation technique to date. On the downside, however, additional data preparation or conversion may be required to allow for meaningful analysis when using CNNs for omics data in this way. Therefore, we call on research that investigates the following:

RQ A-2: Which data preparations are necessary to apply XAI-enhanced CNNs to omics data?

Moreover, choosing a suitable NN for a given use case can be very challenging, given that they can be laid out for diverse data types and applications. This is especially problematic in the omics sciences, where the data themselves and possible outputs may not be fully comprehensible (i.e. underlying biological processes are unclear). We, therefore, pose the question:

RQ A-3: Which XAI approaches are the most promising for NNs applied to omics data to understand the underlying biological processes?

### Add post-hoc analysis to transparent models

In principle, transparent models such as Bayesian networks or decision trees do not require post-hoc explainability as the models themselves are interpretable. However, transparent models can also get very complex when dealing with vast amounts of high-dimensional data like omics data. Therefore, applying additional post-hoc explanations to, in principle, already transparent models can help reduce complexity and improve the understandability of these models and their outputs. For example, our results show that feature relevance is especially used in statistics-based approaches (25.0%) and gradient boosting (78.3%), approaches that are regarded transparent. Most rule mining approaches are considered transparent (70.0%), but post-hoc explainability methods like architecture modifications (16.7%) are used as well. For most transparent models, we also found some studies using visual explanations. For example, four studies visualized their statistics-based approach, while three studies added visual explanations on top of their rule mining model. Therefore, it might be worth investigating extant and novel transparent models with post-hoc explainability techniques to improve understandability. This investigation should also include guidelines on best-suited post-hoc explainability methods depending on the different ML models. Therefore, we pose the exemplary RQ:

RQ B-1: How can post-hoc explanations improve the understandability of transparent models for omics data?

Visual explanations are some of the most helpful post-hoc explainability methods to improve human understandability, as they allow for the interpretation of vast amounts of data in a single (or a select few) images. In our review, visual explanations account for 52 of 250 post-hoc approaches (20.8%) and are applied to 14 different AI methods. Additionally, many studies having other primary explainability methods (such as feature relevance or architecture modification) nonetheless use visual explanations as an auxiliary measure. For example, Patel-Murray *et al*. [99] visualize their clustering results, although their focus lies on model simplification. Similarly, results and performance graphs could also be seen as visual explanations. We argue that all AI methods, and in particular transparent models, do benefit from visualizing the input, model or output and thus propose the following exemplary RQ:

RQ B-2: How can visual explanations aid the analysis of omics data with transparent AI models?

### Use simplification and local explanations for post-hoc analysis of omics data

Within our literature set, we only identified a limited number of studies that employed simplification (*n* = 14) and local explanations (*n* = 16) as their explainability methods. This finding is not surprising as implementing such methods is often more complex than, for example, feature relevance.

Simplification, for one, not only requires a good understanding of how AI methods can be simplified, but researchers must also be adept with the use case and omics data at hand. While random forests are the most popular (*n* = 5), one other group of methods seemingly suited for simplification is NNs, as attempting to reduce the number of hidden layers and nodes should be a natural first step. However, we only saw five out of 184 publications applying simplification to NNs. While DNNs usually improve predictive performance, they often lack interpretability due to their depth and black box characteristics [9, 10]. Exactly those characteristics often hinder the simplification of NNs. Nonetheless, simplifying DNNs into shallow ANNs could maintain sufficient predictive performance while increasing interpretability. This is especially relevant for omics data, as simplifying network structures often allows for more biological interpretability by unveiling how certain processes are connected. For instance, simplification could be introduced by iteratively splitting the network into subnetworks that fulfill specific sub-tasks. Warrell *et al*. [61] apply this simplification approach to cancer-type detection, but it should be applicable to other use cases as well. Hence, we pose:

RQ C-1: How can NNs be simplified to improve the transparency of omics data analysis?

Similarly, local explanations offer increased explainability by explaining only parts of the model. While this does not reduce model complexity, it allows researchers to investigate smaller parts at once. This is especially helpful for imaging tasks. For example, the LIME framework (Local Interpretable Model-Agnostic Explanations) can highlight the most important regions contributing to a CNN’s output decision [100]. Hence, we propose the following RQ:

RQ C-2: How can DL models (such as CNNs) for omics data be explained through local explanations?

### Develop new interpretable models for omics data and adapt existing post-hoc explainability methods

Although AI has been a prominent research topic for several decades now, XAI research for omics data has only recently gained momentum. When dissecting the XAI models used by publication year, we see that almost all studies from 2010 to 2014 in our review followed an interpretable model approach. Starting in 2015, however, there has been a steady increase in post-hoc explainable approaches, outnumbering the interpretable approaches since 2018 and with nearly twice as many approaches in 2022 (31 interpretable versus 61 explainable, cf. Supplementary Material 3). These observations are in line with previous findings on XAI research in general, highlighting the fast increase of XAI-focused studies and increasing post-hoc explainability approaches [10]. These post-hoc explainability approaches, especially feature relevance approaches like SHAP, are most often applied to DL techniques established in practice. This may be the case because it is often easier to add additional post-hoc explainability methods than to develop new interpretable models, for example, through architecture modification. However, most DL techniques are only sparsely established in healthcare practice. Therefore, it remains unanswered how these post-hoc explainability approaches could be included in practice at clinical sites. Hence, we propose the following RQ:

RQ D-1: Which measures are necessary to construct post-hoc explainable models with omics data that are deployable in healthcare practice?

Especially for non-transparent models (e.g. NNs or SVMs), post-hoc explainability models are more common (73.6%). Nonetheless, there are first attempts in literature, such as Young and Lu [101], who propose to modify the architecture of a DNN into a so-called redundant input NN, allowing them to achieve partial interpretability of the hidden layers. Their approach allows them to investigate the genomic alterations in cancer cell signaling. Modifying model architectures may offer a higher potential of fully incorporating existing knowledge and understanding the underlying biological processes. To achieve this, however, researchers must develop new interpretable models altogether or achieve interpretability through alteration (i.e. architecture modification or simplification) of existing DL methods. Therefore, we propose:

RQ D-2: How can current non-transparent models be modified (e.g. through architecture modification) into novel interpretable models?

### Combine different explainability methods

When choosing a post-hoc approach, usually multiple methods are tested for fit with the given use case. In fact, we presume that many researchers included in our study have tested different explainability methods but only presented the most suitable ones. Occasionally, we found some authors using multiple explainability methods, especially when dealing with multiple AI methods. However, we are not aware of many instances where multiple post-hoc explainability methods are combined (e.g. merged or executed in sequence) but are rather used separately. Certain frameworks such as DeepShap combine different explainability methods, for example, by first calculating Shapley values (i.e. feature relevance) and then visualizing them onto an image (i.e. visual explanation). Our review showed that using multiple AI methods is one of the most common approaches (*n* = 57). Some of these studies create performance-improved multi-model pipelines, a strategy we propose could also be applied to multiple explainability techniques (e.g. [72, 102]). With first promising approaches in that direction, it might be worth investigating how different explainability methods can be combined to improve the human understandability of black box models. Consequently, we pose the RQ:

RQ E-1: How can different explainability methods be combined for AI-based omics data analysis?

### Apply explanations by example and text explanations to omics data

While our mapping mainly includes four types of post-hoc explainability methods (i.e. feature relevance, simplification, local explanation, visual explanation), the original coding based on Barredo Arrieta *et al*. [10] includes two further post-hoc methods, namely, explanation by example and text explanations. The very low representation of these methods, however, is not surprising to us, as both approaches are also seldom found in other research domains. Nonetheless, research has already shown the applicability and advantages of both explanations by example (e.g. [103]) and text explanations (e.g. [104]).

Applying explanations by example to omics data could, for example, help experts determine the correct classification or prediction of a model by selecting single data examples that represent a certain class, thus providing an understanding of how the model classifies certain input data. With this theoretical applicability and potential benefits to omics data analysis, we pose the following RQ:

RQ F-1: How can explanations by example improve post-hoc analysis of omics data?

Similarly, the semantic properties of text explanations make output results understandable in human-readable terms and may thus help doctors and practitioners not familiar with the inner workings of an AI method interpret its results. Additionally, text explanations may also aid omics data researchers in producing human-readable output explaining the underlying biological processes. Based on the rapid improvements in large language models, the generation of text explanations could be facilitated. In fact, our review did include two recent approaches applying text explanations [105, 106], demonstrating the applicability for omics data. Therefore, we pose:

RQ F-2: How can text explanations improve post-hoc analysis of omics data?

### Investigate XAI for novel omics data

Our review also highlighted that more novel data types like 3D-genomic data (*n* = 3) or certain gene mutation data (*n* = 14) appear to be analyzed less frequently by means of XAI. We found some omics data types not to be analyzed with the help of XAI in the literature at all, such as cellular lipids, cellular carbohydrates, ribosomal and messenger RNA sequence or genotyping data. Especially the latter is gaining momentum, with direct-to-consumer genetic testing becoming popular for personal health [107]. With XAI showing promising results for many omics data types, research should continue investigating XAI not only for existing use cases and data but also, especially for novel omics data. Hence, we pose:

RQ G-1: How can XAI foster analysis of novel omics data?

### Explore the role of XAI for omics data in decision-making processes

Lastly, within our study, we noticed that contemporary research on XAI for omics data is mostly dedicated to the implementation of diverse XAI approaches. Only four studies in our reviewed literature followed a mixed methods approach, with the rest focusing merely on design and implementation (cf. the research approach and research method columns in Supplementary Material 1). While these are highly valuable and desired research efforts, we currently lack an understanding of how XAI approaches actually afford (bio)medical experts to gain new insights and how they impact their decision-making in omics use cases. There is a nascent stream of literature that investigates experts’ cognitive processes in joint human–AI decision-making settings (e.g. [108, 109]). However, these studies usually take the stance of opaque, black box AI models providing recommendations to experts. They thus mostly neglect XAI methods’ potential to increase the understandability and transparency of black box AI models (e.g. through providing insights into the underlying biological processes [14]) and how this might impact human cognition and decision-making processes. Recognizing this gap in extant research, we raise the following research question:

RQ H-1: How does XAI afford (bio)medical experts to gain new insights, and how does it impact experts’ decision-making in omics use cases?

### Limitations and contributions

Our research is not without limitations. First, the systematic mapping method is subjective to some degree and depends on the facets and categories deduced during the keywording and data extraction steps. We strived to minimize subjectivity by including multiple researchers in the analyses. Second, we only considered peer-reviewed research articles and preprints from select sources. This excluded non-scientific publications such as white papers and blog posts, which can often entail more recent findings on XAI and omics data. Nevertheless, we are confident that, together with the inclusion of preprints, our systematic mapping study includes a broad range of XAI applications for omics data.

Our study makes important contributions to research and practice. First, we contribute to the different and interdisciplinary research streams of XAI for omics data by synthesizing the knowledge and contributions of the various research communities to this field of research. In doing so, we summarize and highlight the main findings of current XAI implementations for omics data found in extant literature. Moreover, our results can help make researchers aware of other fields with similar interests, transmit previously isolated knowledge, bridge the gap and foster collaboration between these distinct research communities. Second, we utilize our overview to deduct and present eight directions for future research on XAI for omics data. These research directions outline open and interesting future issues in the field that, based on our review, need to be addressed to move the field forward. We also provide exemplary RQs per research direction, which may serve as concrete starting points for future research. Third, we developed a systematic mapping scheme, which can be utilized to classify and map future implementations of XAI for omics data. Our classification scheme can aid developers as an orientation as to which approaches have already been tried and how their XAI implementation may contribute to omics research. With slight alterations of the classification scheme to the data-related dimensions, it can also be easily adapted to other healthcare-related fields where XAI is of interest.

## CONCLUSION

In this study, we investigated extant literature on the application of XAI for omics data by means of a systematic mapping study. We identified 405 relevant publications; developed a coding scheme with 10 facets focusing on AI, XAI and omics data aspects; and mapped all studies accordingly. We investigated frequent patterns in the combination of AI methods, omics data and explainability methods. Subsequently, we deduced eight future research directions for research on XAI for omics data as a contribution to research and practice. With our mapping study, we, therefore, shed light onto this interdisciplinary and quickly growing research stream, seeking to bridge the gap between isolated islands of research on the topic. Further, this review may support the transfer of knowledge between the omics sciences and XAI research and thus enable a more targeted progression of the literature on XAI for omics data.

**Key Points**

- Systematic mapping study of 405 papers with a 10-facet classification scheme addressing the used AI and explainability methods as well as the underlying omics data.
- We analyzed the AI methods used for omics data and the explainability method per AI method, from which we derived eight research directions and a total of 14 exemplary research questions for future investigation.
- Most approaches in our sample applied explainability methods on neural networks, so we call for additional research on the application of explainability methods on other AI models or data types.
- Our sample showed only a few approaches applying simplification, local explanations or text explanations. Therefore, future research should investigate the use of previously unused explainability methods.
- With mostly post-hoc explainable approaches present in our sample, we argue for more transparent approaches or a combination of both.

## Supplementary Material

S1_Literature_list_bbad453

S2_Development_of_the_classification_scheme_bbad453

S3_Additional_results_bbad453

S4_Glossary_of_abbreviations_bbad453

## Data Availability

The data underlying this article are available in the article and in its online supplementary material.

## Author Biographies

**Philipp A. Toussaint** is a research associate at the Karlsruhe Institute of Technology, Karlsruhe, Germany and the HIDSS4Health—Helmholtz Information and Data Science School for Health, Karlsruhe/Heidelberg, Germany. His research interests include the application of explainable artificial intelligence (AI), biomedical data analysis and direct-to-consumer genetic testing. Currently, he is researching the application of explainable AI methods for omics data.

**Florian Leiser** is a research associate at the Karlsruhe Institute of Technology, Karlsruhe, Germany. His primary research interests include informed machine learning, health informatics and federated learning. In his recent work, he investigates the inclusion of expert knowledge in medical image annotation.

**Scott Thiebes** is a postdoctoral researcher at the Karlsruhe Institute of Technology, Karlsruhe, Germany, where he also received his PhD. His primary research interests include emerging technologies in healthcare, information privacy and security and persuasive health technologies. In his recent research, he explores the design and implications of trustworthy AI in healthcare settings.

**Matthias Schlesner** is a professor of biomedical informatics, data mining and data analytics at the Faculty of Applied Computer Science and Medical Faculty, University of Augsburg, Augsburg, Germany. His primary research interests are the development and application of methods for the computer-assisted analysis of biomedical data.

**Benedikt Brors** is a professor and head of the Division of Applied Bioinformatics at the German Cancer Research Center (DKFZ), Heidelberg, Germany and the National Center for Tumor Diseases, Heidelberg, Germany. His primary research interest is combining computer science with statistical and biomedical methods to improve the understanding of cancer development and the diagnosis as well as therapy of cancer.

**Ali Sunyaev** is a professor at the Karlsruhe Institute of Technology, Karlsruhe, Germany. His research interests are reliable and purposeful information systems within the scope of critical infrastructures, cloud computing services, information security solutions, trustworthy AI, auditing/certification of IT and innovative health IT applications. His research work accounts for the multifaceted use contexts of digital technologies with research on human behavior affecting IT applications and vice versa.
