# Supplementary material for: Explainable artificial intelligence for omics data: a systematic mapping study
Source: Brief Bioinform. 2023 Dec 18;25(1):bbad453. doi: 10.1093/bib/bbad453 (PMC10729786; doi:10.1093/bib/bbad453)
Supplement: S2_Development_of_the_classification_scheme_bbad453 [file s2_development_of_the_classification_scheme_bbad453.pdf]

S2: Development of the classification scheme

This document provides additional insights into the development and further explanations of the presented classification scheme. As outlined in our main manuscript, we followed the five-step procedure by Petersen [1], whereas the development of the classification scheme is mainly conducted during step 4. Hence, this supplement elaborates on the keywording process, meaning the extraction of relevant aspects that formed the facets and contained categories of our developed classification scheme.

Keywording using abstracts

According to Petersen [1], a classification scheme consists of several facets, which each entail a number of possible categories. A facet, in this sense, represents an aspect of interest in the underlying data, for example, the research approach of a study. The categories of a facet then represent the specific data instances. For example, our paper uses a systematic mapping approach (category) for its research approach (facet). Categories of a facet should be mutually exclusive, so only one category per facet should apply to a single data instance. Further, while there might be additional possible categories, usually, the classification scheme only includes those found in the underlying data. In other words, classification schemes could also be thought of as a taxonomy with dimensions and characteristics.

During the fourth step of our research approach, keywording using abstracts, we sampled a subset of 45 papers out of the 405 included studies (11.1%). Three researchers analyzed the abstracts and searched for keywords, which were then used to deduct relevant facets and categories for the topic of interest. Deducing facets and categories this way has the advantage of familiarizing oneself with the underlying data. Additionally, the deduced categories are relevant to the data, which may not be the case when deducing categories from theory.

Due to the interdisciplinary nature of our study, facets should represent the three main aspects of this study, namely artificial intelligence (AI) models, explainability methods, and omics data.

Therefore, we formed ten facets in a discussion among all authors. Additionally, we also collated three facets for meta-information, such as the scientific field the respective outlet can be attributed to.

Artificial intelligence facets

To account for the technical view, we first investigated the *AI method* used in each paper. This facet should represent the underlying AI model. It may either be transparent (e.g., decision trees or linear/logistic regression) or non-transparent (e.g., deep neural networks). To better differentiate the different forms of deep learning methods, we distinguished between simple 3-layer artificial neural networks (ANNs), i.e., one input layer, one hidden layer, and one output layer, and multi-layer deep neural networks (DNNs). If the study allowed for the deduction of the specific type of neural network, it was classified as such instead. The found types include convolutional neural networks (CNNs), graph neural networks (GNNs), recurrent neural networks (RNNs), transformers, and variational autoencoders. In total, we identified 18 categories for this facet, including a category for the use of multiple AI methods (see Table S2-1).

Another facet regarding the technical view is the *AI task*. We differentiate between classification, clustering, and prediction tasks as well as any combination of them. The classification task is further divided into binary classification (i.e., something is present or not) and multiclass classification (i.e., selection from multiple possible labels for one data point). The only combination of tasks we identified in our sample is a combination of prediction and classification tasks, resulting in five categories for this facet.

Explainability facets

As the second group of facets, we gathered four facets regarding the use of explainable artificial intelligence (XAI) in our coding scheme. All four facets are inspired by Barredo Arrieta, Díaz-Rodríguez [2] but are essential to our understanding of XAI (see Table S2-2).

Table S2-1. Overview of Artificial intelligence-related facets.

Facet	Explanation	Categories
AI method	Learning architecture/ algorithm used	• Artificial neural network (ANN)<br>• Bayesian network<br>• Clustering algorithm<br>• Convolutional neural network (CNN)<br>• Decision tree<br>• Deep neural network (DNN)<br>• Generalized linear model (GLM)<br>• Gradient boosting<br>• Graph neural network (GNN)<br>• Linear/Logistic regression<br>• Multiple AI methods<br>• Random forest<br>• Recurrent neural network (RNN)<br>• Rule mining<br>• Statistics-based approach<br>• Support vector machine (SVM)<br>• Transformer<br>• Variational autoencoder
AI task	Type of task the AI model performs	• Classification - multiclass<br>• Classification - binary<br>• Clustering<br>• Prediction<br>• Prediction and Classification

The first XAI facet describes the *model approach*. In this facet, we analyzed all papers as to whether they present a new XAI model approach or utilize an existing one. Therefore, this facet only contains two categories (existing model or new model).

The *XAI model* facet differentiates whether the paper uses an interpretable (i.e., inherently transparent) model or explainable (post-hoc) model on top of a traditional AI method. This distinction is not always trivial since the application of some explainability methods can also transform previously non-transparent models into transparent ones. These difficulties were assessed by frequent discussions among the researchers and double coding in especially challenging cases.

The applied *explainability method* is the third XAI-related facet we captured. We followed the eight XAI methods specified by Barredo Arrieta, Díaz-Rodríguez [2] but identified no applications of explanations by example in our sample. Therefore, our coding scheme contains only seven categories. These explainability methods are outlined in more detail in Table S2-3.

Table S2-2. Overview of XAI-related facets.

**Facet**	**Explanation**	**Categories**
Model approach	Whether a new model is proposed	• Existing model<br>• New model
XAI model	Whether the model is directly interpretable or post-hoc explainable	• Explainable (post-hoc) model<br>• Interpretable model
Explainability method	Which explainability technique the XAI model uses	• Architecture modification<br>• Feature relevance<br>• Local explanation<br>• Simplification<br>• Text explanation<br>• Transparent model<br>• Visual explanation
XAI generalizability	Whether the XAI approach can be transferred to other AI methods	• Model-agnostic<br>• Model-specific

The final facet in this group is *XAI generalizability,* where we differentiate between model-agnostic approaches that could be applied to other AI methods and model-specific approaches that only suit this specific AI method. For example, we coded the adoption of SHapley Additive exPlanations (SHAP) values as model-agnostic because they can be applied to all AI methods. On the contrary, most architecture modifications were regarded as model-specific since they strongly rely on the AI method used.

Omics and medical-related facets

The third group entails four medical facets (see Table S2-4). The first medical facet is the *omics field* from which the data emerged. For this, we created an initial list of the most common omics studies (see also omics related part of our search string for this initial list). Given our dataset, however, this resulted in only nine different categories, with genomics being the most present, followed by transcriptomics.

The second facet regarding the medical field is the specific type of *omics data* used as input for the models of each paper. These categories were gathered for each paper and, later on,

discussed amongst the researchers on how to align them. In our sample, this resulted in ten categories.

Table S2-3. Overview of included explainability methods.

Explainability method	Explanation
Architecture modification	The architecture of an otherwise non-transparent model is altered to achieve a transparent model. For example, current approaches try to modify the layer architecture of neural networks through redundant inputs in every layer to achieve transparency [3].
Feature relevance	Feature relevance methods try to clarify the inner functioning of a model by computing a relevance score for how variables contributed to the output. For example, SHapley Additive exPlanation (SHAP) values can show the marginal contribution of each input data point to the output prediction, thus allowing humans to understand which data points were important to the prediction.
Local explanation	Local explanations aim to segment the model to provide a solution of subspaces relevant to the whole model. This way, humans can understand an AI decision by understanding subparts of the whole model. The most famous framework for this is LIME (Local Interpretable Model-Agnostic Explanations) [4].
Simplification	A new and simpler model with similar performance is implemented based on the original non-transparent model. Often, rules are extracted to achieve transparency. For example, random forests can sometimes be simplified into a single decision tree to achieve transparency.
Text explanation	Text explanations bring explainability by generating human-understandable text to explain the results from the model. For example, an image classification model may additionally describe what it sees in the picture to explain its classification decision. Text explanations also include methods generating symbols that outline the functioning or decision strategy of the model.
Transparent model	The model by itself is inherently interpretable and thus has the ability to explain or to provide the meaning in understandable terms to a human without additional methods. Typical models include linear/logistic regression, clustering algorithms, decision trees, rule mining models, Bayesian models, or general additive models.
Visual explanation	Visual explanations visualize the model's behavior or output. Usually, this requires a dimensionality reduction to allow for a human-understandable image/graph. While applicable to any AI model, they are most often used for image classification tasks. The probably most famous visualization techniques include heatmaps and saliency maps.

The final two facets gathered in our coding scheme are *medical use case* and *medical field*. Categories for the medical use case were gathered similarly to the categories of *omics data*. While coding the paper, the researchers identified the statements of the authors, which were later grouped into 24 suitable categories, ranging from binding site prediction and cancer type classification to gut microbiota analysis and neurological disorder prediction. The *medical field* dimension is derived from the list of North American medical specialties [5]. While this list currently comprises 52 medical specialties, many only rarely involve omics data, such as general surgery. Hence, we only identified 17 categories across our sample.

Table S2-4. Overview of omics and medical-related facets.

Facet	Explanation	Categories
Omics field	Concerned discipline from the omics field	• Epigenomics<br>• Genomics<br>• Metabolomics<br>• Microbiomics<br>• Multiple<br>• Pharmacogenomics<br>• Proteomics<br>• Toxicogenomics<br>• Transcriptomics
Omics data	Input data for the XAI model	• 3D-Genomic<br>• DNA sequence<br>• Gene expression<br>• Gene mutation<br>• Microbiomic<br>• Multi-omics<br>• Proteomic<br>• RNA sequence<br>• sc-RNA sequence<br>• SNP
Medical use case	The specific medical use case that the XAI model aims to solve	• Binding site prediction<br>• Biomarker identification<br>• Cancer detection<br>• Cancer type classification<br>• Cell type prediction<br>• Chromosome structure detection<br>• Disease risk prediction<br>• Disease severity prediction<br>• DNA pattern identification<br>• DNA variant detection<br>• Drug response prediction<br>• Gene expression patterns<br>• Gene regulation modeling<br>• Gut microbiota analysis

Facet	Explanation	Categories
		• Heredity prediction<br>• Interaction networks inference<br>• miRNA detection<br>• Neurological disorder prediction<br>• Pathogenicity prediction<br>• Phenotype association<br>• Protein localization prediction<br>• Protein structure prediction<br>• Splice site prediction<br>• Survival prediction
Medical field	The medical field in which the XAI model is applied	• Allergy and immunology<br>• Cardiology<br>• Clinical laboratory sciences<br>• Dermatology<br>• Dietetics<br>• Endocrinology<br>• Forensic medicine<br>• Gastroenterology<br>• Infectious disease<br>• Medical research<br>• Neurology<br>• Oncology<br>• Ophthalmology<br>• Oral and maxillofacial surgery<br>• Pathology<br>• Psychiatry<br>• Rheumatology

Additional facets

We finally captured three facets that should detect more general meta-information about the analyzed manuscripts. Table S2-5 shows an overview of all collected supplementary facets and respective categories.

First is the general *research approach* followed by a study. The specific topic of interest led to the inclusion of only two categories, namely design and mixed methods. The second facet, *research method*, captures the method of each paper. Due to the nature of the researched field, all included papers implemented their approaches. Some of the included papers also describe their conceptualization process in detail, which is why we added the second manifestation. The third supplementary facet is the *scientific field* to which the outlet is

attributed to. Based on the outlet's specification, we classified the attributed scientific field with regard to the Australian and New Zealand Research Classification [6].

Table S2-5. Overview of supplementary facets.

Facet	Explanation	Categories
Research approach	The approach of the paper	• Design<br>• Mixed methods
Research method	The conducted method of the paper	• Implementation<br>• Implementation and Concept
Scientific field	The scientific field the outlet is attributed to	• Biomedical and clinical sciences<br>• Engineering<br>• Information and computing sciences<br>• Multidisciplinary<br>• Sciences (biological, chemical, mathematical)

References

1. Petersen KF, Robert; Mujtaba, Shahid; Mattsson, Michael. Systematic Mapping Studies in Software Engineering. In: Visaggio G. B., Maria Teresa; Linkman, Steve; Turner, Mark (ed) Proceedings of the 12th International Conference on Evaluation and Assessment in Software Engineering. Italy: BCS Learning & Development Ltd., 2008, 68–77.
2. Barredo Arrieta A, Díaz-Rodríguez N, Del Ser J et al. Explainable Artificial Intelligence (XAI): Concepts, taxonomies, opportunities and challenges toward responsible AI, Information Fusion 2020;58:82-115.
3. Young JD, Lu X. Revealing the impact of genomic alterations on cancer cell signaling with a partially transparent deep learning model, bioRxiv 2020:2020.2005.2029.113605.
4. Ribeiro MTS, Sameer; Guestrin, Carlos. "Why Should I Trust You?": Explaining the Predictions of Any Classifier. In: Proceedings of the 22nd ACM SIGKDD International Conference on Knowledge Discovery and Data Mining. San Francisco, California, USA, 2016, p. 1135–1144. Association for Computing Machinery.
5. Specialties ABoM. Specialty and Subspecialty Certificates. https://www.abms.org/member-boards/specialty-subspecialty-certificates/ (26 July 2023, date last accessed).

6. Statistics ABo. Australian and New Zealand Standard Research Classification (ANZSRC). https://www.abs.gov.au/statistics/classifications/australian-and-new-zealand-standard-research-classification-anzsrc/2020 (26 July 2023, date last accessed).
