# Supplementary material for: Explainable artificial intelligence for omics data: a systematic mapping study
Source: Brief Bioinform. 2023 Dec 18;25(1):bbad453. doi: 10.1093/bib/bbad453 (PMC10729786; doi:10.1093/bib/bbad453)
Supplement: S3_Additional_results_bbad453 [file s3_additional_results_bbad453.pdf]

# S3: Additional results

This document provides additional results for our conducted systematic mapping study. We will present the distinction between explainable and interpretable models over the past years and two additional bubble charts that might provide insightful results contemplating our review.

## Explainable models

Figure S3-1 shows an overview of the identified papers in our study per publication year. The total for each year is the same compared to Figure 3 of the main manuscript. Here, however, we distinguish between interpretable (i.e., transparent) models and post-hoc explainable models.

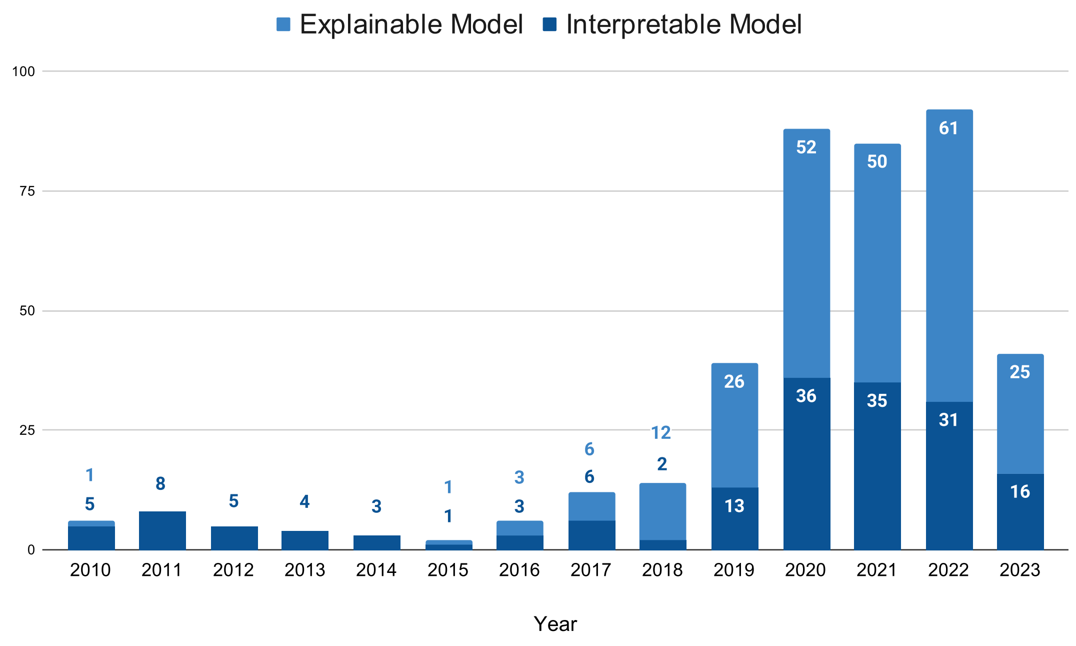


**Figure S3-1.** Number and explainability approach of publications per year.

In our sample, most studies before 2015 are considered interpretable. The only exception to this is the study by Stiglic, Rodriguez [1] published in 2010, who apply feature relevance to a random forest approach. Starting in 2015, we identified 1 explainable model. Thereafter, explainable models gained ever more interest and surpassed interpretable models (n=12 vs.

n=2) in 2018. In 2022, we identified nearly twice as many explainable (n=61) than interpretable approaches (n=31). Simultaneously, peaking in 2020, the number of interpretable models has been decreasing in recent years, from 36 in 2020 to 31 manuscripts in 2022. This indicates an increased interest in post-hoc explainable models, which is in line with the frequent use of non-transparent models such as neural networks in recent years.

## Explainability method per omics data

Within our review, we analyzed the combinations of the applied artificial intelligence (AI) method and used omics data, as well as the AI method and explainability method. In this section, we want to discuss the third combination of explainability method and omics data (see Figure S3-2). Since the descriptive findings for these dimensions are already explained in the manuscript, here, we will discuss only the relationship between both.

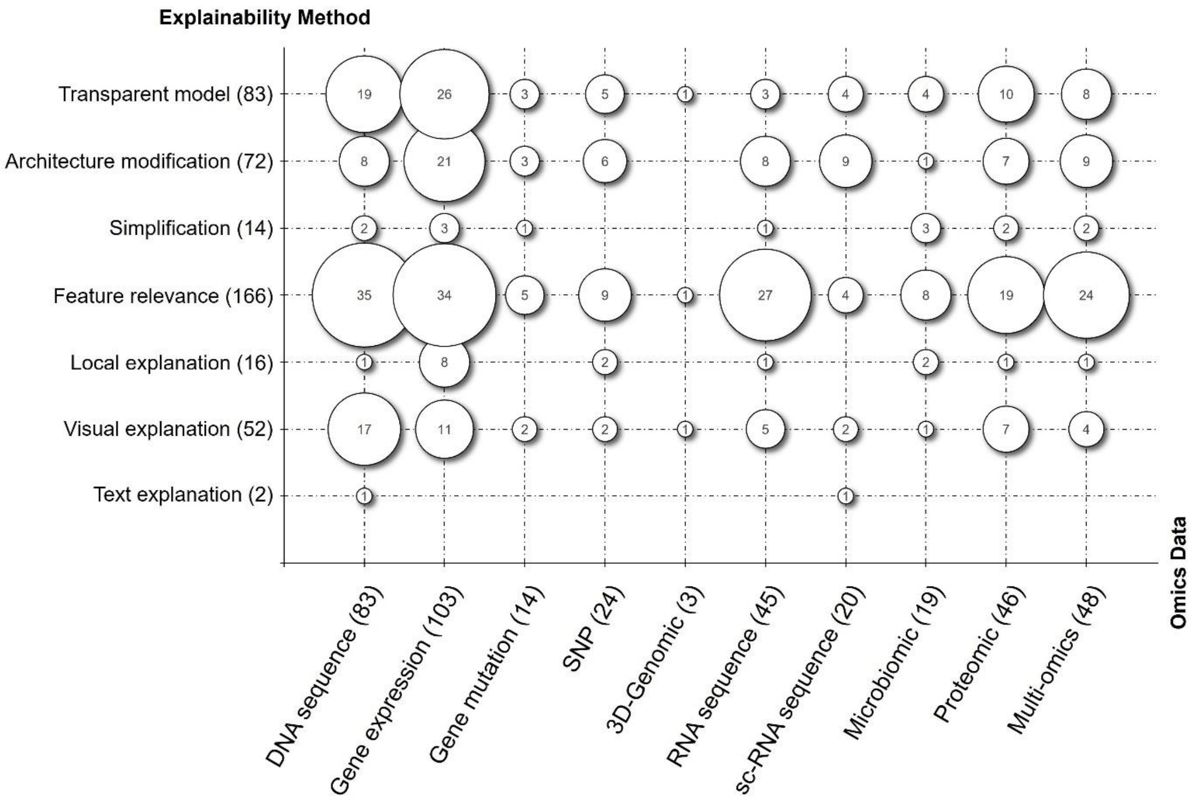


**Figure S3-2.** Bubble chart of applied explainability method and used omics data.

When comparing the explainability method applied to the omics data, most combinations are present in our dataset. With feature relevance being the most frequent explainability method,

it is used for all categories of omics data, most dominantly for DNA sequence, gene expression, and RNA sequence data. For example, Pai and Bader [2] apply patient similarity as a feature relevance to cancer detection tasks. Transparent models and visual explanations are also applied to all omics data types. Architecture modifications are also used across almost all categories except for 3D-genomics, where we only included three papers in our sample.

Only DNA sequence data is analyzed with every single explainability method. If we exclude text explanations, where we only identified two manuscripts, this list expands to include gene expression, RNA sequence, proteomic, and multi-omics data. With the equal distribution across the categories, our analysis yielded only a few frequent combinations. The overall most frequent combination of categories in the explainability method and omics data chart is feature relevance for DNA sequence data (n = 35), followed by feature relevance for gene expression (n=34), and feature relevance for RNA sequence data (n=27). Overall, feature relevance is used in more than 40% of the approaches. Besides feature relevance, the most prevalent combination is transparent models on gene expression data (n=26), for example, to detect obesity [3].

## AI method and underlying medical field

We included additional facets besides the three discussed in the main manuscript and the section above. While most facets remained unelusive, we present the combination of the applied AI method and the underlying medical field in Figure S3-3.

Most included studies were conducted in the domains of medical research (n = 164) or oncology (n = 121). Across those two categories, the overall distribution of the AI methods is represented. We emphasize here that 65.3% of the approaches using convolutional neural networks (CNNs) conduct medical research like Ghanbari and Ohler [4], who predict RNA binding sites with attribution maps. We also found that support vector machines (SVMs) are more frequently used for oncology data (58.3%), such as Minhas, Asif [5], who analyze context-aware feature mappings on multiple cancer-related data sets.

Many frequent approaches are also used in other medical fields, such as gradient boosting approaches used in clinical laboratory sciences and deep neural networks in psychiatry. Rule mining approaches are used across nine different medical fields, indicating that they are a very versatile approach.

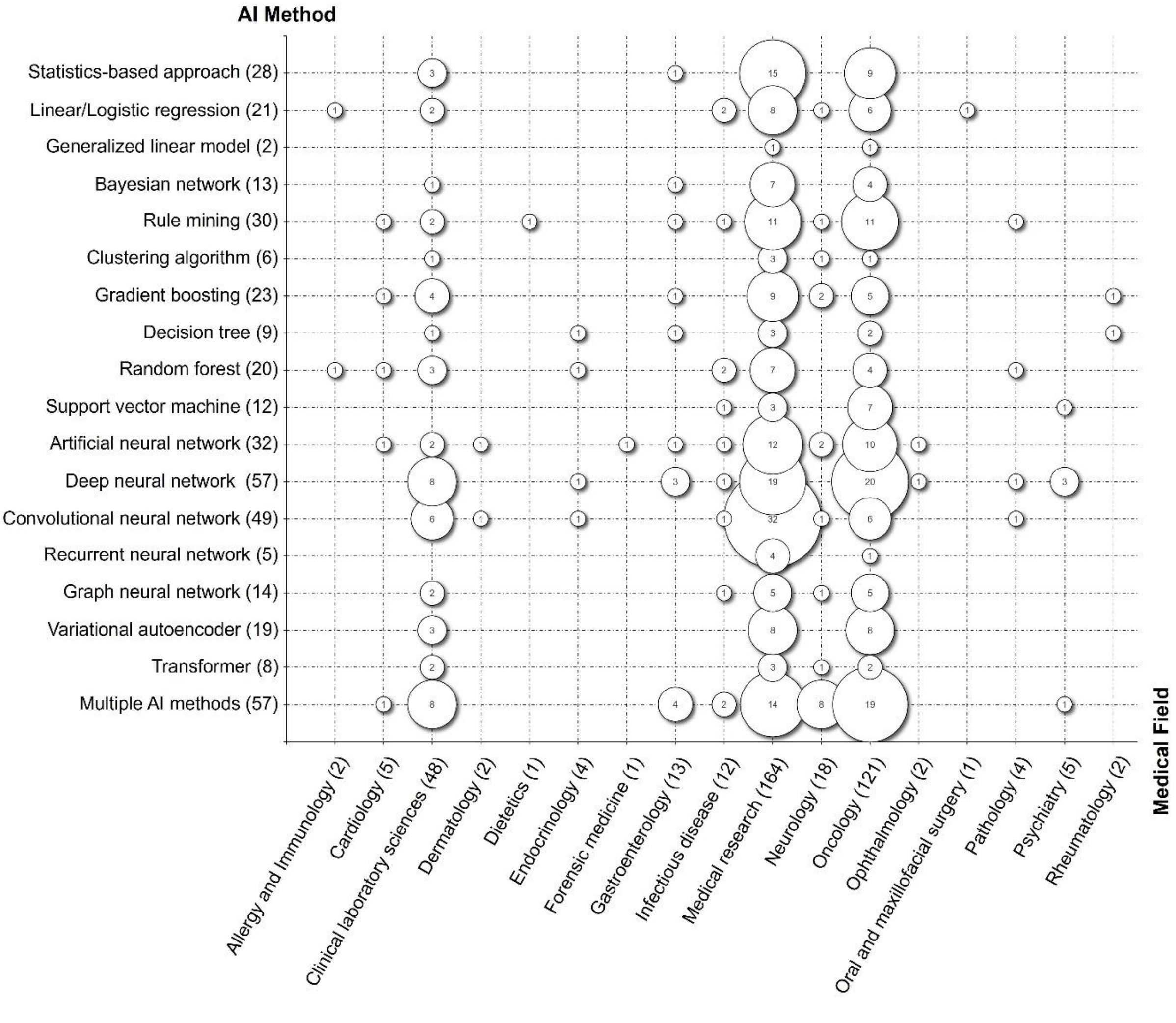


**Figure S3-3.** Bubble chart of AI method and underlying medical field.

# References


1. Stiglic G, Rodriguez JJ, Kokol P. Finding optimal classifiers for small feature sets in genomics and proteomics, Neurocomputing 2010;73:2346-2352.
2. Pai S, Bader GD. Patient Similarity Networks for Precision Medicine, J Mol Biol 2018;430:2924-2938.

3. Anguita-Ruiz A, Segura-Delgado A, Alcala R et al. eXplainable Artificial Intelligence (XAI) for the identification of biologically relevant gene expression patterns in longitudinal human studies, insights from obesity research, PLoS Comput Biol 2020;16:e1007792.

4. Ghanbari M, Ohler U. Deep neural networks for interpreting RNA-binding protein target preferences, Genome Res 2020;30:214-226.

5. Minhas F, Asif A, Arif M. CAFE-Map: Context Aware Feature Mapping for mining high dimensional biomedical data, Comput Biol Med 2016;79:68-79.
