# Supplementary material for: Explainable artificial intelligence for omics data: a systematic mapping study
Source: Brief Bioinform. 2023 Dec 18;25(1):bbad453. doi: 10.1093/bib/bbad453 (PMC10729786; doi:10.1093/bib/bbad453)
Supplement: S4_Glossary_of_abbreviations_bbad453 [file s4_glossary_of_abbreviations_bbad453.pdf]

S4: Glossary of abbreviations

This document provides an alphabetic overview of abbreviations found in the main manuscript and supplementary material (See Table S4-1).

Table S4-1. Overview of used abbreviations.

**Abbreviation**	**Full term**
AI	Artificial Intelligence
ANN	Artificial Neural Network
CNN	Convolutional Neural Network
DL	Deep Learning
DNA	Deoxyribonucleic Acid
DNN	Deep Neural Network
GLM	Generalized Linear Model
GNN	Graph Neural Network
LIME	Local Interpretable Model-Agnostic Explanations
miRNA	Micro Ribonucleic Acid
ML	Machine Learning
NN	Neural Network
RNA	Ribonucleic Acid
RNN	Recurrent Neural Network
RQ	Research Question
Sc-RNA	Single-Cell Ribonucleic Acid
SHAP	SHapley Additive exPlanations
SNP	Single Nucleotide Polymorphism
SVM	Support Vector Machine
XAI	Explainable Artificial Intelligence
